# The first record of the *Dendrodoris arborescens* (Collingwood, 1881) (Gastropoda, Nudibranchia) in the Yellow Sea (Dalian, Liaoning, China)

**DOI:** 10.3897/BDJ.14.e199742

**Published:** 2026-08-12

**Authors:** Ying Tian, Wenjia Li, Yida Han, Yuyoumin Wen, Yifei Liu, Linxuan Cai, Zhenlin Hao

**Affiliations:** 1 Dalian Ocean University, Dalian, China Dalian Ocean University Dalian China

**Keywords:** Dendrodorididae, distribution range, Heterobranchia, Yellow Sea

## Abstract

**Background:**

The nudibranch genus *Dendrodoris* Ehrenberg, 1831 (Mollusca, Gastropoda, Nudibranchia, Dendrodorididae) comprises a group of marine gastropods commonly known as "doris"nudibranchs. Members of this genus are characterised by their soft, flattened bodies, lack of radular teeth and reliance on sponge prey for nutrition. *D. arborescens* (Collingwood, 1881) originally was described from the Haitan Strait in the East China Sea, subsequent records having been reported from South Korea, Japan, Australia and the Philippines.

**New information:**

This study documents the first record of the dendrodorid nudibranch *Dendrodoris arborescens* (Collingwood, 1881) from the northern Yellow Sea, China. This study is based on twenty specimens collected from the lower intertidal zone at Heishijiao, Dalian, China (38.864987N, 121.548425E) between December 2023 and September 2024.

The species identification was confirmed through morphological examination and molecular analysis of the COI gene sequence. This discovery extends the known northern distributional limit of the species by approximately 800 km from Jeju Island, South Korea and represents the first record of both the species and the genus *Dendrodoris* Ehrenberg, 1831 in northern Chinese waters. The potential role of the Kuroshio Current extension in facilitating long-distance dispersal of planktotrophic larvae is discussed, along with the significance of this finding for updating the marine biodiversity inventory of northern Chinese waters.

## Introduction

The genus *Dendrodoris* Ehrenberg, 1831, a shell-less heterobranch clade, is characterised by considerable morphological plasticity. The genus is widely distributed across the Indo-Pacific, Atlantic and Australian Regions and currently comprises 46 recognised species (Burn 2006, Chow et al. 2022, Gosliner et al. 2023a, MolluscaBase 2026). Species within this genus, which lack spicules and a radula (Alder and Hancock 1864, Young 1969), often exhibit striking, yet variable body colouration and patterns, even within a single species (Rose 1985, Valdés et al. 1996). While these features are important for taxonomy, their variability, coupled with overall anatomical similarity, has historically led to significant confusion and misidentification amongst species (Brodie et al. 1997, Brodie and Calado 2006, Zenetos et al. 2017, Galià-Camps et al. 2022).

The *D. arborescens* (Collingwood, 1881) typifies these taxonomic challenges. Originally described from the Haitan Strait in the East China Sea, off Fujian Province (Collingwood 1881), subsequent records have been reported from the South China Sea, South Korea, Japan, Australia and southern Africa (Chichvarkhin 2016, Park et al. 2019, Chow et al. 2022, Lee and Min 2022, Gosliner et al. 2023b). Historically, its identity was obscured by two widespread, morphologically similar congeners, being considered either a dark form of *D. fumata* (Rüppell & Leuckart, 1830) or a synonym of *D. nigra* (W. Stimpson, 1855) (Brodie et al. 1997, Burn 2006). However, its larval development provided critical diagnostic evidence: *D. arborescens* exhibits planktotrophic veliger larvae with two distinct features - a large cephalopedal region that replaces the operculum and cannot be retracted into the shell and an early shedding of the shell during the pelagic phase (Rose 1985, Brodie and Calado 2006, Cai et al. 2025). Based on unique developmental traits, Goddard (2005) hypothesised that *D. arborescens* represented an independently evolving lineage (Hirose et al. 2014). This was later confirmed by Brodie and Calado (2006), who formally reinstated it as a valid species, based on a synthesis of external morphology, colour pattern, gill structure and larval characteristics. The Yellow Sea, a marginal sea of the western Pacific with relatively low water temperatures, hosts a molluscan fauna dominated by warm-temperate species. While a limited number ofnudibranchs are known from this region, no species of *Dendrodoris* has been previously recorded (Qi et al. 1989, Zhang et al. 2016). Although *D. arborescens* was first described from Chinese waters, confirmed records within China have been scarce and historically confined to southern regions (Liu 2008). Its presence was only recently documented in Hong Kong (Chow et al. 2022).

This study reports the first occurrence of *Dendrodoris arborescens* in the northern Yellow Sea. This finding significantly extends the known northern geographical limit of the species and contributes an important update to the documentation of marine biodiversity in northern Chinese waters.

## Materials and methods

### Sample collection

**Figure 1.**
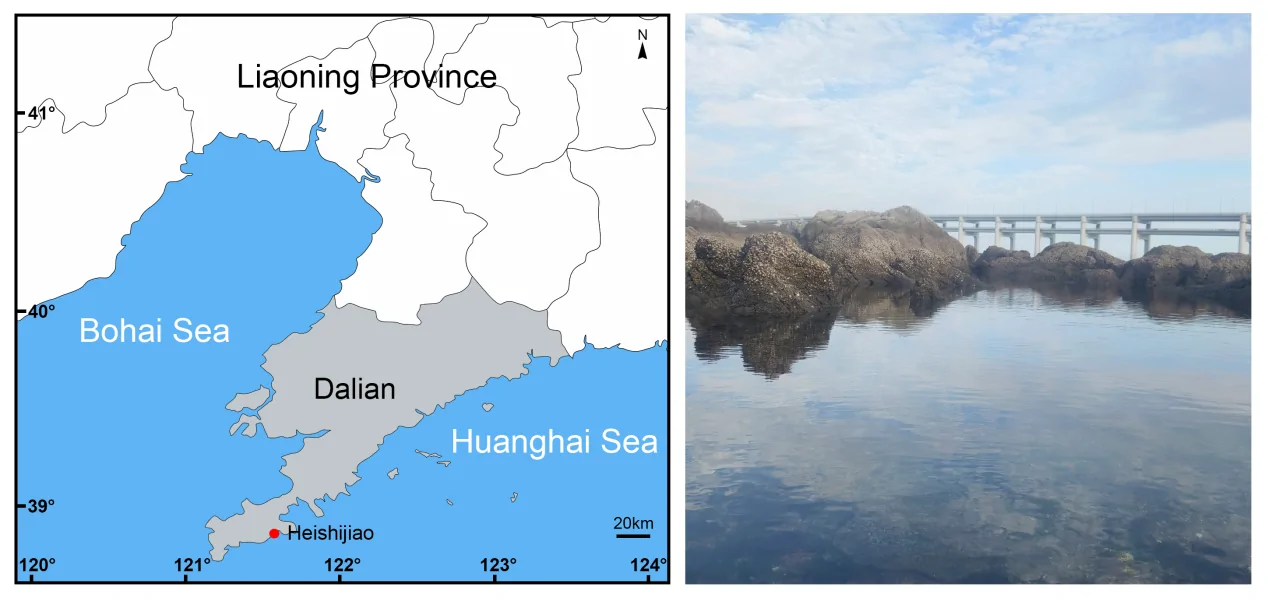
Location (left) of the collection site (right) at Heishijiao, Dalian, Liaoning Province, China, in the northern Yellow Sea.

Twenty specimens of *Dendrodoris arborescens* were collected from the lower intertidal zone at Heishijiao, Dalian, Liaoning Province, China (38.86499°N, 121.54843°E) (Fig. 1) Collections were made during low tide between December 2023 and February 2024, with additional specimens found in September 2024. The first specimen was collected on 28 December 2023. Specimens were found on the underside of rocks, co-occurring with the gastropod *Gigahomalopoma nocturnum* (A. A. Gould, 1861) and the polyplacophoran *Acanthochitona rubrolineata* (Lischke, 1873). Specimens were photographed in situ and examined alive in the laboratory. Identification was based on diagnostic external morphological characteristics as established in recent taxonomic literature (Brodie and Calado 2006). Specimens were preserved in 95% ethanol and deposited in the reference collection of the Key Laboratory of Mariculture & Stock Enhancement in the North China Sea, Dalian Ocean University，with registration numbers DLOUDA01–DLOUDA20 (DLOU: Dalian Ocean University; DA: *Dendrodoris arborescens*).

### Phylogenetic Analysis

Total genomic DNA was extracted from foot tissue of three specimens using the TIANamp Marine Animal DNA Kit (Tiangen Biotech, Beijing, China) following the manufacturer's protocol. Approximately 20 mg of tissue was finely minced and incubated overnight at 56°C in 200 μl of lysis buffer. DNA concentration and purity were assessed using a NanoDrop 2000 spectrophotometer (Thermo Fisher Scientific, USA), with A₂₆₀/₂₈₀ ratios ranging from 1.8 to 2.0. The mitochondrial cytochrome c oxidase subunit I (COI) gene was amplified using the universal primer pair LCO1490/HCO2198. PCR reactions were performed in a 25 μl volume containing 12.5 μl of 2× Master Mix (Tiangen), 1 μl of each primer (10 μM), 1 μl of template DNA and 9.5 μl of ddH₂O. Thermal cycling conditions were: initial denaturation at 94°C for 3 min; 35 cycles of 94°C for 30 s, 50°C for 30 s and 72°C for 1 min; final extension at 72°C for 10 min. PCR products were verified on 1.5% agarose gels and sent for bidirectional sequencing at Sangon Biotech (Shanghai, China).

To maximise taxonomic representation, published mitochondrial gene sequences of Heterobranchia were retrieved from NCBI GenBank. Given the limited availability of complete mitochondrial genomes for this group, representative COI sequences from Dendrodorididae and closely-related taxa were selected to construct the phylogenetic dataset. Multiple sequence alignment was performed using MAFFT. Poorly-aligned and ambiguous positions were subsequently trimmed using Gblocks. The trimmed alignment was then partitioned by codon position: first plus second positions formed one partition and third positions a separate partition.

Phylogenetic inference was conducted using the Maximum Likelihood (ML) method in IQ-TREE. For each partition, the automated model selection strategy was applied to identify the optimal substitution model independently. Tree searches were subsequently performed under the best-fitting models to obtain the ML topology. Node support was evaluated using bootstrap re-sampling with 1000 replicates to assess branch support values and evaluate the robustness of key topological relationships. Given the elevated evolutionary rate in certain smooth-bodied species within the genus *Dendrodoris*, the alignment was visualised using terminal trimming to accommodate alignment difficulties.

## Taxon treatments

### Dendrodoris arborescens

(Collingwood, 1881)

4653EB8B-C694-5C08-A054-F8F19996335C

#### Materials

- **Type status:** Other material. **Occurrence:** sex: hermaphrodite; lifeStage: adult; occurrenceStatus: present; occurrenceID: FE12EE2B-B75F-56DB-AFC9-625802A8138B; **Taxon:** taxonID: urn:lsid:marinespecies.org:taxname:533877; scientificNameID: urn:lsid:marinespecies.org:taxname:533877; originalNameUsageID: urn:lsid:marinespecies.org:taxname:533879; nameAccordingToID: https://doi.org/10.18195/issn.0313-122x.69.2006.119-126; scientificName: *Dendrodoris arborescens*; parentNameUsage: Dendrodorididae O'Donoghue, 1924 (1864); originalNameUsage: *Doridopsis arborescens* Collingwood, 1881; kingdom: Animalia; phylum: Mollusca; class: Gastropoda; order: Doridida; family: Dendrodorididae; genus: *Dendrodoris* ; taxonomicStatus: accepted; **Location:** country: China; countryCode: CN; stateProvince: Liaoning; county: Dalian; locality: Heishijiao; locationRemarks: Heishijiao, Shahekou District, Dalian City, Liaoning Province, China (GPS: 38.86499°N, 121.54843°E); verbatimCoordinates: 38.86499, 121.54843; decimalLatitude: 38.864987; decimalLongitude: 121.548425; **Event:** habitat: Low intertidal, under rocks; **Record Level:** language: en; basisOfRecord: LivingSpecimen; **Material Entity:** associatedSequences: http://www.ncbi.nlm.nih.gov/nuccore/PV759423

#### Description

**Figure 2.**
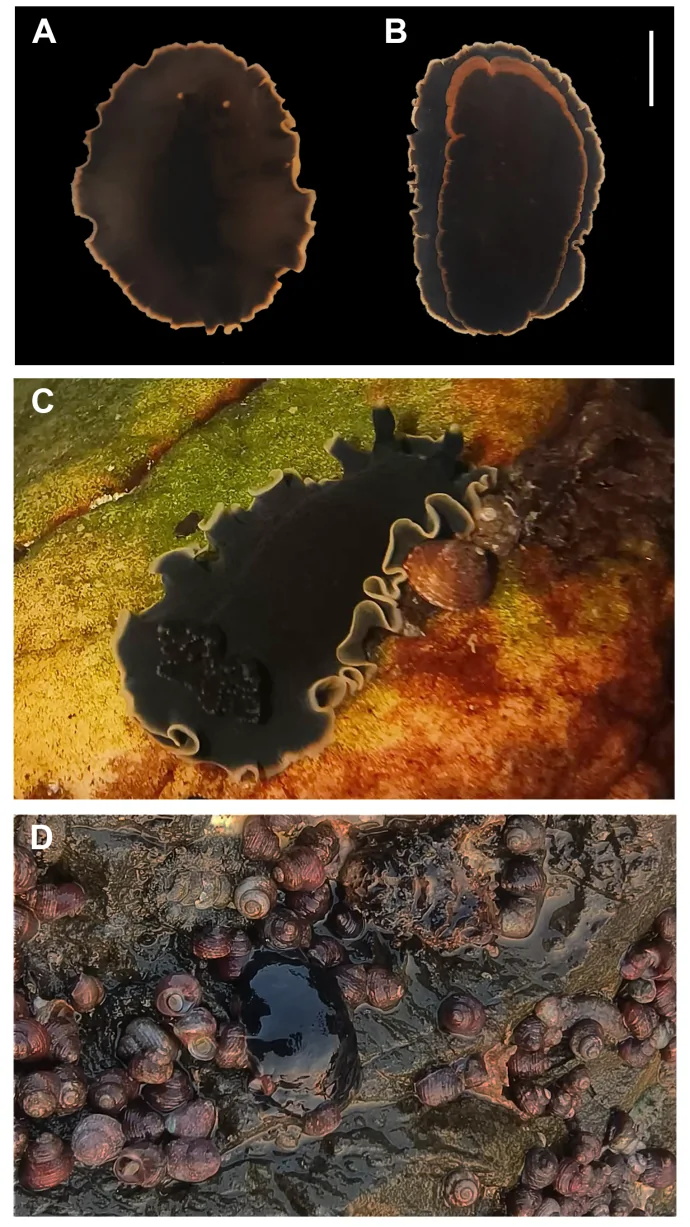
*Dendrodoris arborescens* **A** Dorsal view; **B** Ventral view; **C** Ecological photography; **D** First discovered in Heishijiao, Dalian (2023.12.28), at the bottom of an intertidal rock, living with *Gigahomalopoma nocturnum* (A. A. Gould, 1861) and *Acanthochitona rubrolineata* (Lischke, 1873).

Adult specimens are oval-shaped and relatively low and elongate, with smooth, often undulating margins. All specimens have a black mantle with a smooth surface and a fringing orange marginal band; the foot is grey and the gills and rhinophores are black. The gills are feather-like and arranged in a circle around the anal papilla and the tips of the rhinophores are papillate (Fig. 2).

#### Distribution

*Dendrodoris arborescens* has been reported from China (East China Sea; South China Sea; Yellow Sea), South Korea, Japan, the Philippines，Australia and southern Africa. The present specimens from the Yellow Sea represent the northernmost record of the species.

## Analysis

### Species identification

The collected specimens were identified as *Dendrodoris arborescens* (Collingwood, 1881), based on the diagnostic external morphology: a uniformly jet-black mantle and a distinctive bright orange or yellow marginal band (Fig. 2). These characters are consistent with the original description of *Dendrodoris arborescens* Collingwood, 1881 (Collingwood 1881) (as *Doridopsis arborescens*), as well as with subsequent re-descriptions and records (Brodie and Calado 2006, Park et al. 2019, Chow et al. 2022). NCBI BLAST analysis of the COI sequence (Sayers et al. 2010) revealed 98.4% similarity to sequences attributed to *D. arborescens* in GenBank. However, as most reference sequences in the database are short barcode fragments (approximately 660–700 bp), the query coverage was relatively low (approximately 45%) when compared with the full-length COI sequence (1533 bp) obtained in this study. This limited coverage is attributable to the shorter length of the reference sequences and does not compromise the homology assessment of the aligned region.

**Figure 3.**
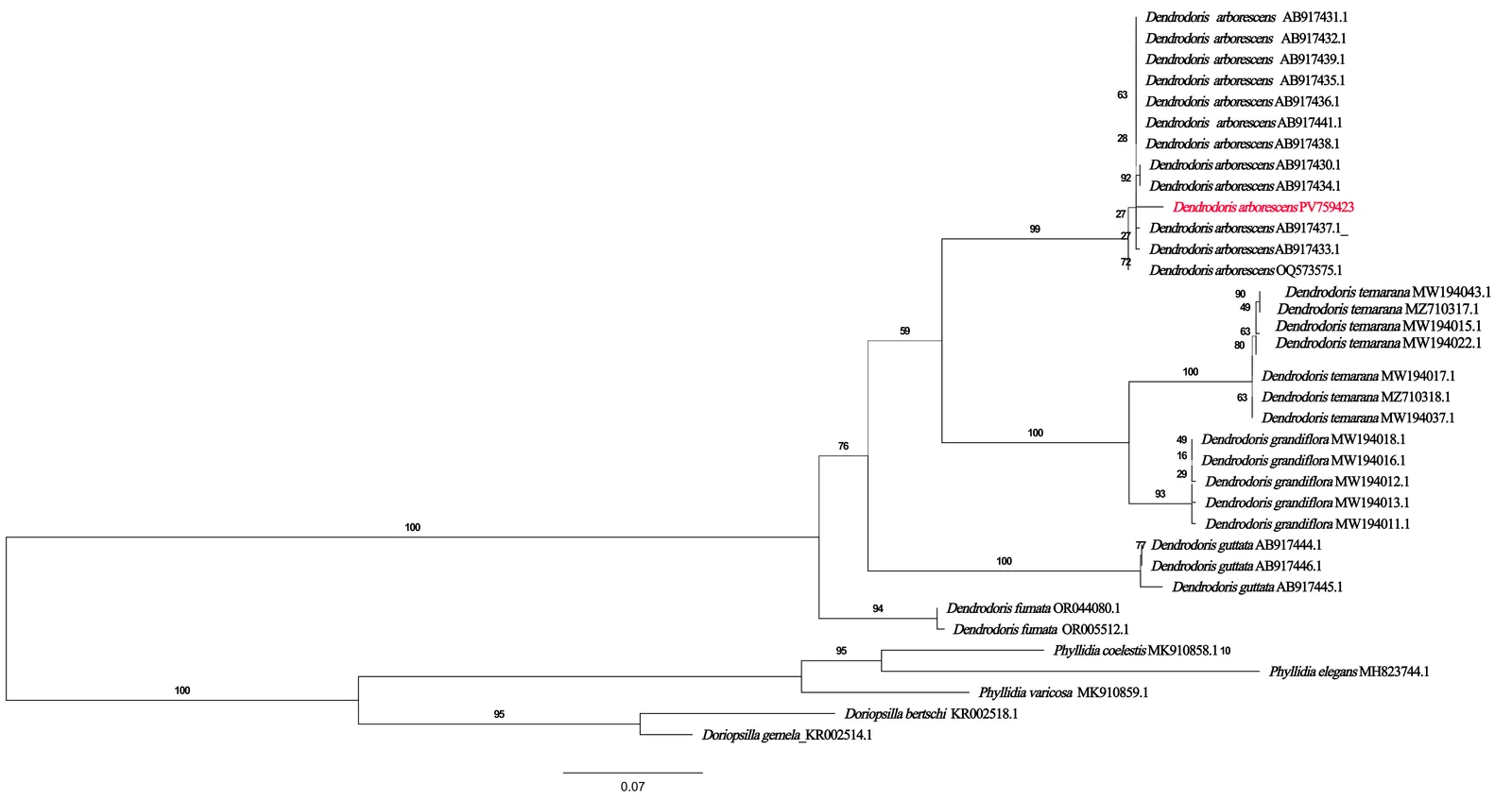
ML tree for species assignment, based on the shared overlapping region of COI (534 bp). The sequence generated in this study, *Dendrodoris arborescens* (PV759423), is highlighted in red.

Maximum Likelihood phylogenetic analysis was performed on a dataset comprising the sequences obtained in this study, those retrieved from BLAST results and sequences from closely-related congeneric species. All COI sequences were mapped to a common reference coordinate system (with the full-length COI sequence PV759423 generated in this study as the reference), yielding an alignment of 1533 bp. The proportion of non-missing data was calculated for each column and the longest continuous window meeting the coverage threshold was selected for ML analysis to maximise the available information while controlling for missing data. In the resulting ML tree, based on the overlapping region (534 bp), the sequence PV759423 clustered with multiple reference sequences of *D. arborescens*, forming a distinct clade separate from closely-related species, thereby confirming the identification of the specimens from Dalian as *D. arborescens* (Fig. 3).

## Discussion

### Taxonomic and biological context

As in other dendrodoridnudibranchs, *D. arborescens* lacks spicules and a radula, relying instead on a highly modified feeding apparatus for suctorial predation on sponges (Alder and Hancock 1864, Young 1969). This morphology is consistent across the family Dendrodorididae, which belongs to the Porostomata clade alongside the Phyllidiidae, although these families are considered polyphyletic, based on recent phylogenetic analyses (Brunckhorst 1993).

The reproductive biology of *D. arborescens* is of particular note. Unlike manynudibranchs exhibiting direct development, *D. arborescens* produces planktotrophic veliger larvae characterised by two distinctive features: a large cephalopedal region that replaces the operculum and cannot be retracted into the shell and early shedding of the shell during the pelagic phase (Rose 1985, Brodie and Calado 2006). These larval characteristics were crucial in clarifying the taxonomic status of this species, which had been historically confused with *D. fumata* (Rüppell & Leuckart, 1830) and *D. nigra* (Stimpson, 1855) due to morphological similarity. The *D. arborescens* specimens from the Yellow Sea were observed to spawn under natural water temperature conditions (Cai 2025), consistent with the reproductive mode documented for this species.

### Biogeographical implications

This discovery represents a significant northwards range extension of approximately 800 km from the nearest previously known locality at Jeju Island, South Korea (Park et al. 2019). It also constitutes the first confirmed record of both the species and the genus *Dendrodoris* Ehrenberg, 1831 in the temperate waters of the northern Yellow Sea and, by extension, northern China. Previous molluscan inventories for this region did not report any *Dendrodoris* species (Qi et al. 1989, Zhang et al. 2016).

The presence of *D. arborescens* in Dalian may be attributed to several non-exclusive factors: (1) the northward-flowing warm Kuroshio Current extension (Jiang et al. 2023), which may transport planktotrophic larvae from established populations in Korea or southern Japan; (2) increased sampling effort in temperate regions; or (3) a previously overlooked resident population that has adapted to local conditions. Given the planktotrophic larval strategy of this species, larval transport by ocean currents remains the most plausible mechanism for its occurrence in the northern Yellow Sea. Alternatively, this record may represent a recent range expansion linked to warming ocean temperatures or the species may have been overlooked due to its cryptic habits and resemblance to other dark-colourednudibranchs.

This finding underscores the importance of continued biodiversity surveys in temperate regions. Further molecular studies using COI gene sequencing, following the protocols established by Hirose et al. (2014), would provide valuable confirmation of the taxonomic identification and insights into population connectivity between the Dalian population and other Indo-Pacific populations.

## Supplementary Material

XML Treatment for Dendrodoris arborescens

## References

[B14181075] Alder Joshua, Hancock Albany (1864). Notice of a Collection of Nudibranchiate Mollusca made in India by Walter Elliot, Esq., with descriptions of several new genera and species. The Transactions of the Zoological Society of London.

[B14181084] Brodie GILIANNE D., Willan RICHARD C., Collins JOHN D. (1997). Taxonomy and occurrence of *Dendrodoris
nigra* and *Dendrodoris
fumata* (Nudibranchia: Dendrodorididae) in the Indo-West Pacific region. Journal of Molluscan Studies.

[B14181093] Brodie G., Calado G. (2006). *Dendrodoris
arborescens* (Collingwood, 1881) (Mollusca: Nudibranchia): larval characteristics reveal a masked porostome species. Records of the Western Australian Museum, Supplement.

[B14181126] Brunckhorst David J. (1993). The systematics and phylogeny of Phyllidiid nudibranchs (Doridoidea). Records of the Australian Museum, Supplement.

[B14181135] Burn Robert (2006). A checklist and bibliography of the Opisthobranchia (Mollusca: Gastropoda) of Victoria and the Bass Strait area, south-eastern Australia. Museum Victoria Science Reports.

[B14181144] Cai L. X., Han Y. D., Jia M. H., Li W. J., Hao Z. L., Tian Y. (2025). The spawn and embryonic development of *Dendrodoris
arborescens* (Collingwood, 1881) (Mollusca: Nudibranchia). Israeli Journal of Aquaculture - Bamidgeh.

[B14181155] Chichvarkhin Anton (2016). Shallow water sea slugs (Gastropoda: Heterobranchia) from the northwestern coast of the Sea of Japan, north of Peter the Great Bay, Russia. PeerJ.

[B14181164] Chow L. H., Yu V., Kho Z., See G., Wang A, Baker D., Tsang L. (2022). An updated checklist of sea slugs (Gastropoda, Heterobranchia) from Hong Kong supported by citizen science. Zoological Studies.

[B14181176] Collingwood Cuthbert (1881). On some new species of Nudibranchiate Mollusca from the Eastern Seas.. Transactions of the Linnean Society of London. 2nd Series: Zoology.

[B14181185] Galià-Camps CARLES, Cervera JUAN LUCAS, Valdés ÁNGEL, Ballesteros MANUEL (2022). Attack on crypsis: Molecular and morphological study of *Dendrodoris* Ehrenberg, 1831 (Mollusca: Gastropoda: Nudibranchia) from the Mediterranean Sea and Northern Atlantic Ocean reinstates *Dendrodoris
temarana* Pruvot-Fol, 1953. Zootaxa.

[B14181203] Goddard J. R. (2005). Ametamorphic direct development in *Dendrodoris
behrensi* (Nudibranchia: Dendrodorididae), with a review of developmental mode in the family. Proceedings of the California Academy of Sciences.

[B14181212] Gosliner T. M., Fahey S. J., Valdés Á. (2023). Indo-Pacificnudibranchs and sea slugs.

[B14181220] Gosliner T. M., Jones G., Beasle L. (2023). Southern African sea slugs.

[B14181228] Hirose Mamiko, Hirose Euichi, Kiyomoto Masato (2014). Identification of five species of *Dendrodoris* (Mollusca: Nudibranchia) from Japan, using DNA barcode and larval characters. Marine Biodiversity.

[B14181237] Jiang Zhibing, Zhu Yuanli, Sun Zhenhao, Zhai Hongchang, Zhou Feng, Yan Xiaojun, Zeng Jiangning, Chen Jianfang, Chen Quanzhen (2023). Enhancement of summer nitrogen fixation by the Kuroshio Intrusion in the East China Sea and Southern Yellow Sea. Journal of Geophysical Research: Biogeosciences.

[B14181259] Lee J., Min D. (2022). A catalogue of molluscan fauna in Korea. The Korean Journal of Malacology.

[B14181268] Liu J. (2008). Checklist of marine biota of China seas.

[B14181276] MolluscaBase MolluscaBase. *Dendrodoris* Ehrenberg, 1831. https://molluscabase.org/aphia.php?p=taxdetails&id=137883.

[B14181284] Park J., Lee Y., Shin Y., Kim T., Park J. (2019). *Dendrodoris
guttata* (Nudibranchia: Dendrodorididae) from Korean waters. Animal Systematics, Evolution and Diversity.

[B14181294] Qi Z., Ma X., Wang Z., Xu F., Dong Z., Li F., Lyu D. (1989). Mollusca of Yellow and Bohai.

[B14181305] Rose R. (1985). The spawn and embryonic development of colour variants of *Dendrodoris
nigra* Stimpson (Mollusca: Nudibranchia). Journal of the Malacological Society of Australia.

[B14181314] Sayers E., Barrett T., Benson D. (2010). Database resources of the National Center for Biotechnology Information. Nucleic Acids Research 39 (suppl_1).

[B14181323] Valdés ÀNGEL, Ortea JESÙS, Ávila CONXITA, Ballesteros MANUEL (1996). Review of the genus *Dendrodoris* Ehrenberg, 1831 (Gastropoda: Nudibranchia) in the Atlantic Ocean. Journal of Molluscan Studies.

[B14181332] Young D. (1969). The functional morphology of the feeding apparatus of some Indo-west-Pacific dorid nudibranchs. Malacologia.

[B14181341] Zenetos A., Chnar . M., Crocetta F. (2017). Uncertainties and validation of alien species catalogues: the Mediterranean as an example. Estuarine, Coastal and Shelf Science.

[B14181350] Zhang S., Zhang J., Chen Z., Xu F. (2016). Mollusks of the Yellow Sea and Bohai Sea.

